# The etiology of exudative cutaneous ulcers in a yaws-endemic community prior to receipt of antimicrobials is similar to that found in communities after mass treatment with azithromycin

**DOI:** 10.1128/msphere.00919-25

**Published:** 2026-02-25

**Authors:** Julie A. Brothwell, Evelyn Toh, Yue Xing, Qunfeng Dong, Linda H. Xu, Lorenzo Giacani, Camila G. Beiras, Oriol Mitjà, Xiang Gao, Stanley M. Spinola

**Affiliations:** 1 Department of Microbiology and Immunology, Indiana University School of Medicine, Indiana University 734638, Indianapolis, Indiana, USA; 2 Department of Medicine, Stritch School of Medicine, Loyola University Chicago 828441, Maywood, Illinois, USA; 3 Department of Medicine, University of Washington 7284, Seattle, Washington, USA; 4 Department of Global Health, University of Washington 7284, Seattle, Washington, USA; 5 Skin Neglected Tropical Diseases and Sexually Transmitted Infections Section, Fight Infections Foundation, University Hospital Germans Trias i Pujol 16514, Badalona, Spain; 6 Universitat de Vic-Universitat Central de Catalunya 16783, Vic, Spain; 7 Department of Medicine, Indiana University School of Medicine, Indiana University 196273, Indianapolis, Indiana, USA; 8 Department of Pathology and Laboratory Medicine, Indiana University School of Medicine, Indiana University 12250, Indianapolis, Indiana, USA; Medical College of Wisconsin, Milwaukee, Wisconsin, USA

**Keywords:** *Arcanobacterium haemolyticum*, cutaneous ulcers, *Haemophilus ducreyi*, *Streptococcus pyogenes*, *Treponema pallidum *subsp. *pertenue*, yaws

## Abstract

Mass drug administration (MDA) of azithromycin (AZ) and case finding and treatment of children with cutaneous ulcers (CUs) have been trialed as strategies to eliminate yaws. Metagenomic sequencing of CU swabs obtained from children 3 to 4 years after the initiation of a yaws elimination campaign on Lihir Island showed enrichment for *Treponema pallidum* subsp. *pertenue* (TPE), *Haemophilus ducreyi* (HD), *Streptococcus pyogenes* (SP), and several anaerobic bacteria. Whether these results were influenced by AZ pressure or are generalizable to other populations is unknown. Here, we performed quantitative PCR (qPCR) for TPE, HD, and SP DNAs and metagenomic sequencing on 260 CU specimens collected from children on New Britain Island, whose inhabitants had not received MDA of AZ. Based on qPCR results, specimens were classified as containing no pathogens, a single pathogen, or multiple pathogens. 31.9% of the specimens contained SP, 28.1% HD, and 25.4% TPE DNAs; mixed infections with two or three pathogen DNAs occurred in 16.5% of cases. No pathogen DNA was detected in 31.5% of the specimens, which were defined as idiopathic ulcers (IUs). In most categories, the same pathogen(s) used to classify the ulcers by qPCR were the most abundant taxa present in the specimens. In IU, the most abundant taxon was *Arcanobacterium haemolyticum*; however, its relative abundance was similar across all groups, suggesting that this organism may adapt to the CU environment rather than represent a cause of IU. These data indicate that TPE, HD, and SP are the primary causes of CU in this untreated population.

**IMPORTANCE:**

Cutaneous ulcers (CUs) affect ~100,000 children annually in tropical regions. After mass drug administration (MDA) of azithromycin (AZ) failed to eradicate yaws, the World Health Organization proposed an integrated disease management strategy to control CU, which emphasizes identifying the causative pathogens in each population. This is critical because organisms associated with CU vary geographically, with *Treponema pallidum* subsp. *pertenue* (TPE), *Haemophilus ducreyi* (HD)*, Streptococcus pyogenes* (SP), and *Leishmania spp*. predominating in different countries. We previously found that TPE, HD, and SP DNAs were enriched in CU specimens from children on Lihir Island in Papua New Guinea (PNG), a population heavily exposed to AZ. Here, we show that these three organisms were also the major pathogens in CU specimens from children on New Britain Island in PNG, whose population had not received MDA of AZ, suggesting that our previous findings are generalizable within PNG and not a consequence of AZ exposure.

## INTRODUCTION

In tropical regions of Africa and the South Pacific islands, ~1%–15% of children have painful exudative cutaneous ulcers (CUs) that until recently were attributed primarily to *Treponema pallidum* subsp. *pertenue* (TPE), the causative agent of yaws (1). Because a single dose of oral azithromycin (AZ) is as effective as injectable benzathine penicillin in the treatment of yaws (2, 3), the World Health Organization (WHO) has advocated for clinical trials to examine whether mass drug administration (MDA) of oral AZ and subsequent biannual case finding and treatment with oral AZ could eliminate yaws in endemic areas. However, molecular diagnostic tests performed on swabs of ulcers before MDA have shown that the etiology of CU is multifactorial, and *Haemophilus ducreyi* (HD) has emerged as a major cause of CU in several countries (4–10). Although single-dose AZ is also effective against HD (11), up to ~20%–30% of ulcers are caused by pathogens other than TPE or HD and may not respond to AZ. For example, prior to MDA of AZ on Lihir Island in Papua New Guinea (PNG), 47% of ulcers had detectable HD DNA, 21% were positive for TPE DNA, and 13% were positive for both HD and TPE DNAs. However, ~20% were negative for both TPE and HD DNA (12), and these were defined as idiopathic ulcers (IUs).

The organisms associated with CU also vary by region. For example, in the Oti region of Ghana, 40% of CUs are positive for *Leishmania* species, 67% for TPE, and 73% for HD DNAs; co-infections with more than one organism occur in 68% of cases, and IUs account for only 8% of cases (9). Thus, defining the etiologies of CU in different regions is critical for designing effective elimination campaigns.

We previously investigated the etiology of IU on Lihir Island by performing metagenomic studies on CU swabs obtained 36, 42, and 48 months after MDA of AZ. Of 275 specimens, 23% were PCR-negative for both TPE and HD DNAs or IU. Both 16S rDNA sequencing and shotgun metagenomic sequencing showed that *Streptococcus pyogenes* (SP) was the most abundant taxon in IU and was detected in ~39% of the IU specimens (13, 14). SP DNA was also more abundant in follow-up ulcers classified as not improved vs. improved, raising the possibility that SP was azithromycin resistant (13). In IU specimens lacking SP DNA, *Criibacterium bergeronii* (a member of the *Peptostreptococcaceae* family) and *Fusobacterium necrophorum* were enriched, suggesting that IU likely has multiple bacterial etiologies (14). Shotgun metagenomic sequencing did not identify enrichment of any viral or eukaryotic pathogens in these specimens (14).

A major limitation of our prior work in defining the causes of IU is that the specimens were obtained after MDA of AZ and biannual case finding and treatment of children with CU. SP could have been highly prevalent prior to MDA, but was reduced by AZ in the follow-up period. Alternatively, SP can readily acquire macrolide resistance, and AZ could have selected for macrolide-resistant SP during the follow-up period, thereby increasing the prevalence of SP. In addition, infections due to *C. bergeronii* and *F. necrophorum* do not respond to AZ; these organisms also could have been selected during the follow-up period. Finally, our prior findings had not been replicated in an AZ-untreated population.

To address these limitations, here we performed molecular diagnostic testing for TPE, HD, and SP DNAs, as well as shotgun metagenomic sequencing, on CU specimens from children in East New Britain Province region of PNG, which is located ~185 km from Lihir Island, and whose inhabitants had not previously received MDA of AZ or intensive case finding and treatment of CU.

## MATERIALS AND METHODS

### Participants and specimen collection

This was a substudy of the project “Repurposing clinically approved drugs for yaws with an insight into the cutaneous ulcer disease syndrome (Trep-AB, ERC-2019-STG 850450).” Because AZ resistance in TPE emerged during AZ-based yaws elimination campaigns (8, 15, 16), alternative oral treatments for yaws needed to be evaluated; the primary study aimed to determine whether linezolid (LZD) could serve as an alternative to AZ to cure yaws. The purpose of the substudy was to identify etiological agents of CU by performing PCR for TPE*,* HD, and SP, and metagenomic sequencing of swabs from children with exudative CU who were being evaluated for eligibility in the primary drug trial.

The participants were children from selected schools in East New Britain who presented with a suspected yaws lesion (e.g., CU or papilloma greater than 1 cm in diameter). Oral consent was obtained to perform a Chembio Dual Path Platform (DPP) Syphilis Screen and Confirm Kit by fingerstick and field (naked eye) examination (17). Cases with a dual-positive DPP test result, which indicates that antibodies to both *Treponema pallidum* subsp. *pallidum* (TP) antigens and non-treponemal (lipoidal) antigens are present, suggesting active yaws infection, were invited to participate in both the primary drug trial and the substudy. Those without a dual-positive DPP result were invited to participate in the substudy. After obtaining written informed consent, an ulcer swab was collected. Children who enrolled in the primary drug trial were randomized to receive oral AZ or LZD; those who enrolled only in the substudy, and those who declined either study, were offered oral AZ.

### CU specimens

All CU specimens were collected using methods described previously (4). In brief, a sterile, dry, Dacron-tipped swab was vigorously rubbed at the base of the ulcer and inserted into a microcentrifuge tube containing 1 mL Buffer ATL (Qiagen). The swab shaft was broken, leaving the swab tip in the tube. The specimens were frozen upon collection and maintained at −20°C until shipment to the Giacani laboratory at the University of Washington, where they were stored at −80°C until use. DNA was extracted from 200 μL of the lesion swab specimen using the DNeasy 96 Protocol (Qiagen, Germantown, MD). The extracted DNA was stored at −80°C until use.

### Amplification reactions

As a control for sample quality, all extracted DNA specimens were first tested for the presence of human DNA by PCR amplification of a portion of the β-globin gene with specific primers (Table 1) as previously reported (18). In brief, 50 μL PCRs containing 5 µL of extracted DNA, 200 µM of a dNTP mix, 1.5 mM of MgCl_2_, 0.8 µM of each primer, and 2U of GoTaq Flexi DNA polymerase (Promega, Madison, WI) were performed. The cycling conditions consisted of an initial denaturation of 3 min at 94°C, followed by 45 cycles (94°C for 1 min, 60°C for 2 min, and 72°C for 1 min) and a final extension step of 10 min at 72°C. Amplification results were verified by agarose gel electrophoresis using an E-gel Electrophoresis apparatus (Thermo Fisher Scientific, Waltham, MA). Specimens with a negative β-globin amplification were re-amplified; if still negative, they were not processed further.

**TABLE 1:** Primers and probes used in this study^*a*^

Organism	Target	Primer or probe	Sequence	Reference	Amplicon size
*H. sapiens*	β-Globin	Hs_Bglob_S	5′-GAAGAGCCAAGGACAGGTAC-3′	(13)	268 bp
		Hs_Bglob_AS	5′-CAACTTCATCCACGTTCACC-3′		
*T. pallidum*	*Tp0105* (*polA*)	polA_S	5′-CAGGATCCGGCATATGTCC-3′	(19)	72 bp
		polA_AS	5′-AAGTGTGAGCGTCTCATCATTCC-3′		
		Probe_TP_polA	5’-6FAM-CTGTCATGCACCAGCTTCGACGTCTT- NFQMGB-3’		
	*TprL*	TprL_S	5′-CTCTGCGCACTGAGAATTGCA-3′	(13)	*T.p. pallidum*: 588bp *T.p. pertenue*: 209bp
		TprL_AS	5′-GCAGTTCGGGTCCTTGCCAA-3′		
*H. ducreyi*	16S rRNA	Hd_16S_S	5′-CAAGTCGAACGGTAGCACGAAG-3′	(20)	439 bp
		Hd_16S_AS	5′-TTCTGTGACTAACGTCAATCAATTTTG-3′		
		Probe_HD_16S	5′-VIC-CCGAAGGTCCCACCCTTTAATCCGA-QSY-3′		
*S. pyogenes*	*speB*	speB_S	5′-AAAGTAGGCGGACATGCCTTTG-3′	(21)	103 bp
		speB_AS	5′-CAAGACGGAAGAAGCCGTCAG-3′		
		Probe_SP_speB	5′-ABY-TCGATGGTGCTGACGGACGT- QSY-3′		

^*a*^ S, sense; AS, anti-sense; NFQMGB: nonfluorescent quencher minor groove binder; QSY: nonfluorescent quencher.

To identify TP, HD, and SP DNAs in the CU specimens, a multiplex TaqMan qPCR was performed based on published assays using the primers and probes listed in Table 1 (13, 18, 20, 21). For the multiplex assay, 2 μL of DNA was used in a 10 μL reaction. Primers and probes were used at a final concentration of 0.5 μM in 1× Fast Advanced TaqMan Master Mix (Thermo Fisher Scientific). Cycling conditions were two initial steps of 2 min each at 50°C and 30 s at 95°C, respectively, followed by 45 cycles of 95°C for 15 s and 60°C for 1 min. Control DNAs for treponemal subspecies were available in the Giacani laboratory. Control DNA for HD was extracted from cultures kindly provided by Dr. Gwendolyn Wood (University of Washington), and control DNA for SP was extracted from cells provided by Dr. Frederick Buckner (University of Washington). Cycle threshold (Ct) cutoff values were determined using standard curves of sextuplets of twofold serially diluted DNA. The Ct cutoff value was based on the experimental analytical sensitivity of the reaction and was defined as the last serial dilution where 100% of the specimens tested positive. Based on this approach, we defined the Ct cutoff for SP to be 30 cycles and 35 cycles for HD and TP. The limits of detection for SP, HD, and TP DNAs were 11 copies, 7 copies, and 6 copies per reaction, respectively. A subset of specimens (*N* = 12) for whom positive amplification was detected above the HD cycle threshold was further characterized by analyzing their amplicon size via gel electrophoresis; if they yielded an amplicon of the expected size (439 bp), they were classified as HD-positive.

Positive amplification reactions for TP DNA were further characterized using qualitative PCR to differentiate TPE DNA from TP DNA based on the *tprL* gene (*tp1031*) (22), which yields a shorter amplicon for TPE strains than for TP strains (Table 1). This amplification was carried out in 50 µL reactions containing 5 µL of sample DNA, 2 U of GoTaq Flexi DNA polymerase, 200 µM of dNTP mix, 1.5 mM of MgCl_2_, 0.8 µM of each primer, and 1× GoTaq Flexi buffer (Promega, Madison, WI) with primers listed in Table 1. Amplicon size was confirmed by electrophoresis on 2% agarose gels. If the size did not match that of the positive controls, amplicons were purified using the QIAquick PCR purification kit (Qiagen) and Sanger sequenced by Azenta Life Sciences using the *tprL* sense primer (Table 1). Specimens yielding results that were not consistent with the TPE *tprL* sequence were deemed negative. The *tprL* qualitative PCR was also performed on a subset of specimens (*N* = 6) for whom amplification was detected slightly above the TP-cycle threshold; if these specimens yielded an amplicon of the expected size (209 bp), they were classified as TPE+.

### Metagenomic sequencing

In all, 265 DNA specimens were shipped to Indiana University-Indianapolis and stored at −80°C. DNA concentrations were quantified on a Qubit 4 Fluorometer (Thermo Fisher Scientific) using the Quant-iT dsDNA Assay Kit, High Sensitivity (Invitrogen).

Shotgun metagenomic sequencing libraries were constructed using the Nextera XT DNA Library Preparation Kit (Illumina, Inc.), according to the manufacturer’s protocol, from 1 ng gDNA. Dedicated lots of reagents were used in library preparation to minimize batch effects. The specimens were divided into five pools; each pool contained two control libraries: one constructed from ZymoBIOMICS microbial community standard II (log distribution) gDNA (Zymo Research catalog no. D6310) and one constructed from *Thermus thermophilus* HB8 gDNA (Takara catalog no. 3071). The pools were sequenced on an Illumina Novaseq 6000 S4 flow cell, and 2 × 150 bp reads were acquired at the Indiana University Center for Medical Genomics Core, Indianapolis, Indiana.

### Sequence processing

Raw sequences were demultiplexed, and human reads were removed and counted using Kraken2, as described previously (14, 23). Non-human reads were then annotated to taxonomic counts using MetaPhlAn3 (24). The libraries yielded 55,880,791 ± 20,624,514 (mean ± SD) reads per sample. After removing human reads, the libraries contained 13,898,303 ± 11,484,295 reads per sample. The average number of microbial reads was 36,462 ± 165,289.

### Data analysis and statistics

The relative abundance of taxa identified by MetaPhlAn3 (24) was normalized using an additive log-ratio (ALR) transformation based on human read counts, as described previously (14, 25). In all pools, the *T. thermophilus* and mock community controls yielded the expected bacterial sequences, confirming that our sequencing and annotation approaches could detect these organisms. Clustering was performed using the R packages cluster, factoextra, and clusterSim. Heatmaps were generated using custom scripts together with the R packages taxize, circlize, ComplexHeatmap, vegan, cluster, clusterSim, and microbiome. The vegan package was also used to calculate α- and β-diversity and to perform non-metric multidimensional scaling (NMDS) based on the Euclidean distance matrix derived from ALR-transformed species abundances. Using the adonis2 function, we also performed a permutational multivariate analysis of variance (PERMANOVA) with 999 permutations. Group comparisons were performed using ANOVA for multiple groups and *t*-tests or Wilcoxon tests for pairwise comparisons. Visualizations were generated using custom scripts with ggplot2 and ggpubr. MetaPhlAn3 does not distinguish between *Treponema pallidum* subsp. *pallidum* (TP) and TPE; however, given the clinical context of this study and the use of qualitative PCR of the *tprL* gene to differentiate TPE from TP (Table 1), metagenomic reads classified as TP likely represent TPE.

## RESULTS

### Demographics of the participants

Of the first 265 specimens collected from participants who enrolled in the primary LZD vs. AZ treatment trial for suspected yaws or the etiology of CU substudy between 20 March and 28 March 2023, five contained no detectable DNA and were excluded from the analysis. Among the remaining 260 participants, 80 had a dual-positive DPP test and were eligible for both the primary treatment trial and the substudy, while 180 did not have a dual-positive DPP test and were eligible only for the etiology of CU substudy. Demographic information of the 260 participants is summarized in Table 2; such information was complete for all 80 DPP-positive participants but was missing for 10 of the 180 DPP-negative participants due to recording errors. Overall, 55.8% of the participants were male, and 92.3% were between the ages of 6 and 18 years old (Table 2).

**TABLE 2:** Demographics of the participants in this study^*c*^

Characteristic	DPP+^*a*^	DPP−^*b*^	Total
Male	39 (48.8)	106 (58.9)	145 (55.8)
Female	41 (51.2)	64 (35.6)	105 (40.4)
1–5 years	6 (7.5)	2 (1.1)	8 (3.1)
6–11 years	40 (50.0)	73 (40.6)	113 (43.5)
12–18 years	33 (41.2)	94 (52.2)	127 (48.8)
18+ years	1 (1.2)	1 (0.6)	2 (0.8)
Data missing	0 (0)	10 (5.5)	10 (3.8)
Total	80	180	260

^*a*^ Number who had a DPP test positive for both *Treponema pallidum* and non-*Treponema pallidum* antibodies.

^*b*^ Number who had a DPP test negative for both *Treponema pallidum* and non-*Treponema pallidum* antibodies.

^*c*^ Values in table are numbers (%).

### Classification of CU specimens by PCR

We used real-time multiplex PCR with pathogen-specific primers to identify CU specimens that contained HD, SP, and TPE DNAs, or none of these DNAs (IU) (Table 3). Of the 260 specimens, 72 (27.7%) contained HD DNA, while 84 (32.3%) and 67 (25.8%) contained SP and TPE DNAs, respectively (Table 3). HD, SP, and TPE were detected as single DNAs in 16.9%, 17.7%, and 16.9% of the CU specimens, respectively (Table 3). Mixed infections with two or three pathogen DNAs were present in 44 (16.9%) of the specimens. No pathogen DNA was detected in 82 (31.5%) of the specimens and was therefore categorized as IU.

**TABLE 3:** qPCR results

DNA detected^*a*^	DPP+^*b*^	DPP–^*c*^	Overall
Distribution of pathogens in ulcers			
HD	14 (17.5)^*d*^	58 (32.2)	72 (27.7)
SP	25 (31.3)	59 (32.7)	84 (32.3)
TPE	31 (38.8)	36 (20)	67 (25.8)
Classification of ulcers by qPCR			
HD	11 (13.8)	33 (18.3)	44 (16.9)
SP	17 (21.3)	29 (16.1)	46 (17.7)
TPE	20 (25)	24 (13.3)	44 (16.9)
HD, SP	0 (0)	21 (11.7)	21 (8.1)
HD, TPE	3 (3.8)	3 (1.7)	6 (2.3)
SP, TPE	8 (10)	8 (4.4)	16 (6.2)
HD, SP, TPE	0 (0)	1 (0.6)	1 (0.4)
None (IU)	21 (26.3)	61 (33.9)	82 (31.5)
Total number of ulcers	80	180	260

^*a*^ HD, *H. ducreyi*; SP, *S. pyogenes*; TPE, *T. pallidum* sub. *pertenue*; IU, idiopathic ulcer.

^*b*^ Number who had a DPP test positive for both *Treponema pallidum* and non-*Treponema pallidum* antibodies.

^*c*^ Number who did not have a dual-positive DPP test.

^*d*^ Values in table are numbers (%).

### Sensitivity and specificity of a dual-positive DPP test

In a previous study, a dual-positive DPP test read in the field had a sensitivity of 74.6% in children whose CU specimens contained TPE DNA by PCR (17). In our study, only 31/80 (38.8%) of the children who had a dual-positive test had CU specimens that contained TPE DNA by PCR (Table 3). A negative DPP test was previously reported to have a specificity of 68.8% (17); in our data set, 144 of 180 children with a negative DPP test lacked TPE DNA by PCR, yielding a specificity of 80% (Table 3). Thus, using PCR as the standard for detecting TPE infection, the DPP test in this trial was not as sensitive as what was previously reported but showed comparable specificity. Differences in screening strategies leading to variation in disease stage and lesion size (8) may have influenced the performance of the DPP test in the current study. Overall, these data indicate that approximately 61% of the children had serologic evidence of past or latent infections with TPE.

### CU microbiomes

Of the 260 specimens, four contained insufficient DNA for library construction; the remaining 256 specimens underwent shotgun sequencing. Based on qPCR results for HD, TPE, and SP, specimens were grouped into eight possible categories: single-pathogen positive, double-pathogen positive, triple-pathogen positive, and triple-pathogen negative or IU. Specimens with ambiguous participant labeling (*N* = 7) and the single specimen that was HD+SP+TPE+ by qPCR were excluded from the analysis. The final analysis included 248 specimens.

**Fig 1:**
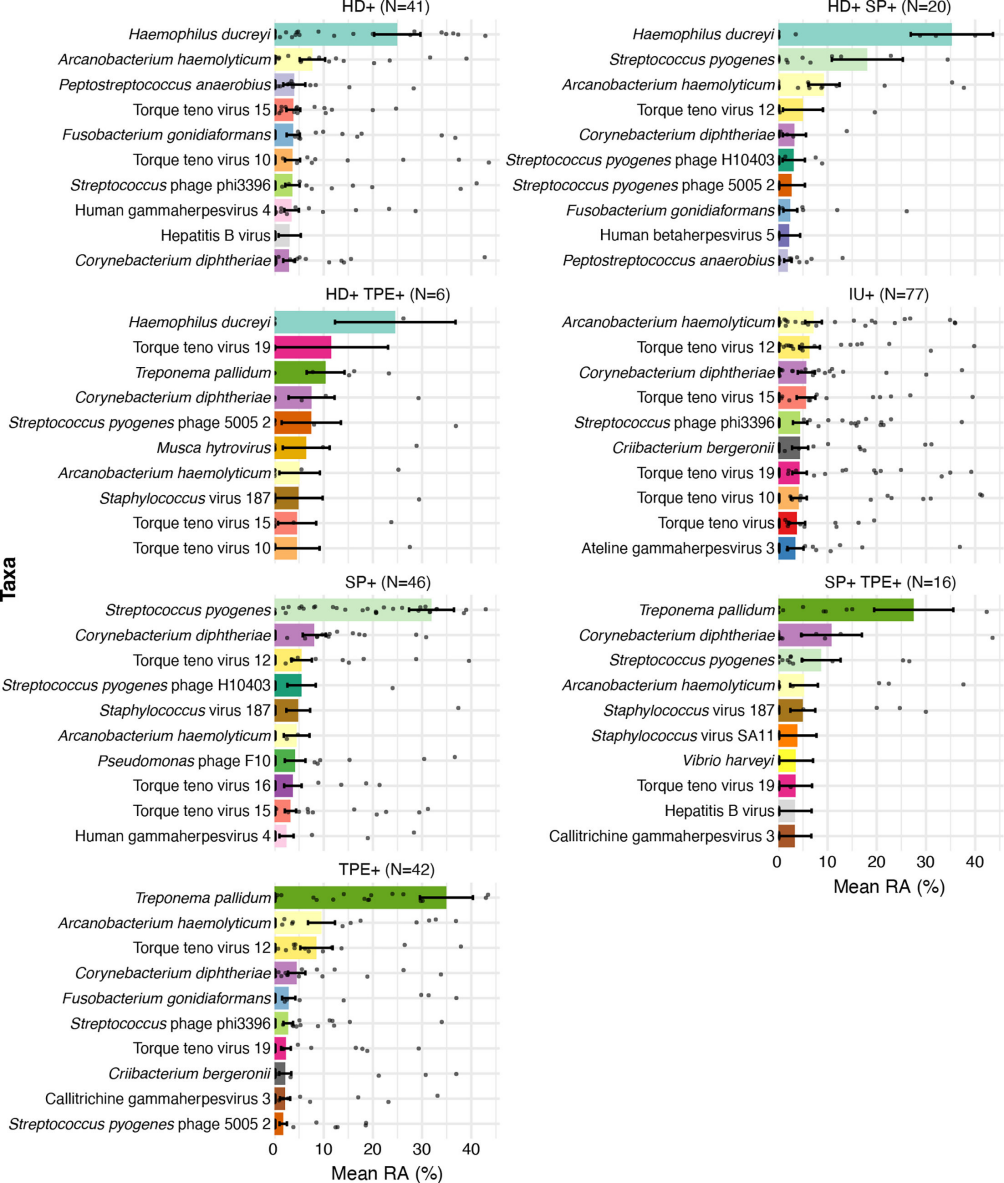
The top 10 most relatively abundant taxa in each CU category. The relative abundances of ALR-transformed MetaPhlAn3 counts for bacteria, viruses, and eukaryotic pathogens were determined for CU that were single- or double-positive for HD, TPE, and/or SP DNAs or negative for all three pathogens (IU) by qPCR. The dots represent the relative abundance of specific taxa in individual samples. CU, cutaneous ulcer; HD, *Haemophilus ducreyi*; TPE, *Treponema pallidum* subsp. *pertenue*; SP, *Streptococcus pyogenes*; IU, idiopathic ulcer; RA, relative abundance.

Metagenomic sequencing identified only one eukaryotic organism, *Malassezia restricta*, which is found on both healthy and diseased human skin (26), in five (2 HD+, 2 HD+TPE+, 1 IU) of the specimens. Data set S1 lists the counts and relative abundance data for all the bacterial and viral species that were identified in each of the seven PCR-defined categories; the ten most relatively abundant organisms in each category are shown in Fig. 1. In six categories, the same pathogen(s) used to classify the ulcers by qPCR were also the most abundant by sequencing, with the exception of the SP+TPE+ category. For the SP+TPE+ ulcers, TP was the most abundant taxon, followed by *Corynebacterium diphtheriae* and then SP. In IU, neither HD nor TP was detected, and SP was detected only at low levels; the most abundant taxon was *Arcanobacterium haemolyticum*. However, the relative abundance of *A. haemolyticum* was similar across all categories (adjusted *P* = 0.302; Data set S1), suggesting that this organism is adapted to the ulcer environment rather than specific to IU.

**Fig 2:**
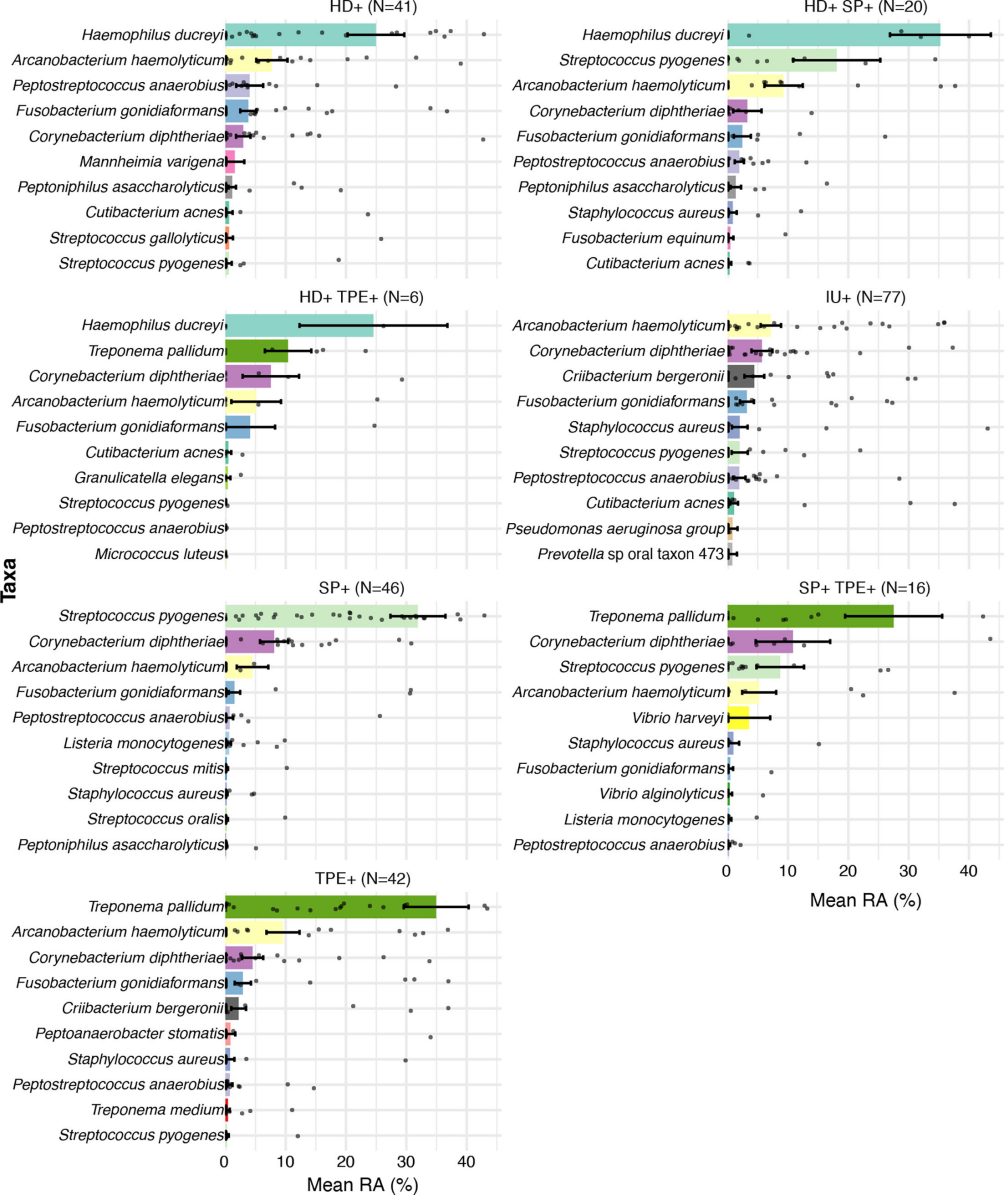
The top 10 most relatively abundant bacterial taxa in each CU category. The relative abundances of ALR-transformed MetaPhlAn3 counts for bacteria were determined for CU that were single- or double-positive for HD, TPE, and/or SP DNAs or negative for all three pathogens (IU) by qPCR. The dots represent the relative abundance of specific taxa in individual samples. CU, cutaneous ulcer; HD, *Haemophilus ducreyi*; TPE, *Treponema pallidum* subsp. *pertenue*; SP, *Streptococcus pyogenes*; IU, idiopathic ulcer; RA, relative abundance.

Various Torque teno viruses were the most abundant viruses detected in each group. Torque teno viruses are ubiquitous in healthy humans, and their roles in disease are unclear (27). Other highly abundant viruses included Callitrichine gammaherpesvirus 3 (a marmoset pathogen) and hepatitis B virus. Two specimens in the IU group (BL_021 and BL_241) contained a high relative abundance of *Molluscum contagiosum*, which, like TPE, can cause papillomas (Data set S1). Because no viruses differed significantly in relative abundance across groups (all *P* > 0.05), we focused on the most relatively abundant bacteria for the rest of the study (Fig. 2).

*A. haemolyticum* and *C. diphtheriae* were among the five most relatively abundant bacterial species across all ulcer categories (Fig. 2). *C. diphtheriae* and *F. gonidiaformans* were detected in all seven categories, and these three species were within the top 10 most relatively abundant bacterial taxa in each group (Fig. 2). In contrast, *C. bergeronii* was restricted to HD+ only, TP+ only, and IU categories (Fig. 2; Table S1).

### The relative abundances of HD, SP, and TPE in the metagenomics data correlate with their qPCR results

We next asked whether the metagenomics data correlated with the qPCR results. For this analysis, we included specimens whose Ct values were less than or equal to the Ct cutoff for each organism. There was a statistically significant inverse relationship between the relative abundances of each pathogen in the metagenomics data and their Ct values as measured by qPCR using the Spearman rank correlation test (TP relative abundance vs. TP Ct: Spearman *r* = −0.8737; HD relative abundance vs. HD Ct: Spearman *r* = −0.8405; SP relative abundance vs. SP Ct; Spearman *r* = −0.7251; all *P* < 0.001). Thus, our data support concordance between sequencing-based relative abundance and PCR-based detection of HD, SP, and TPE in the CU specimens.

### The DPP test may not detect patients with early yaws

**Fig 3:**
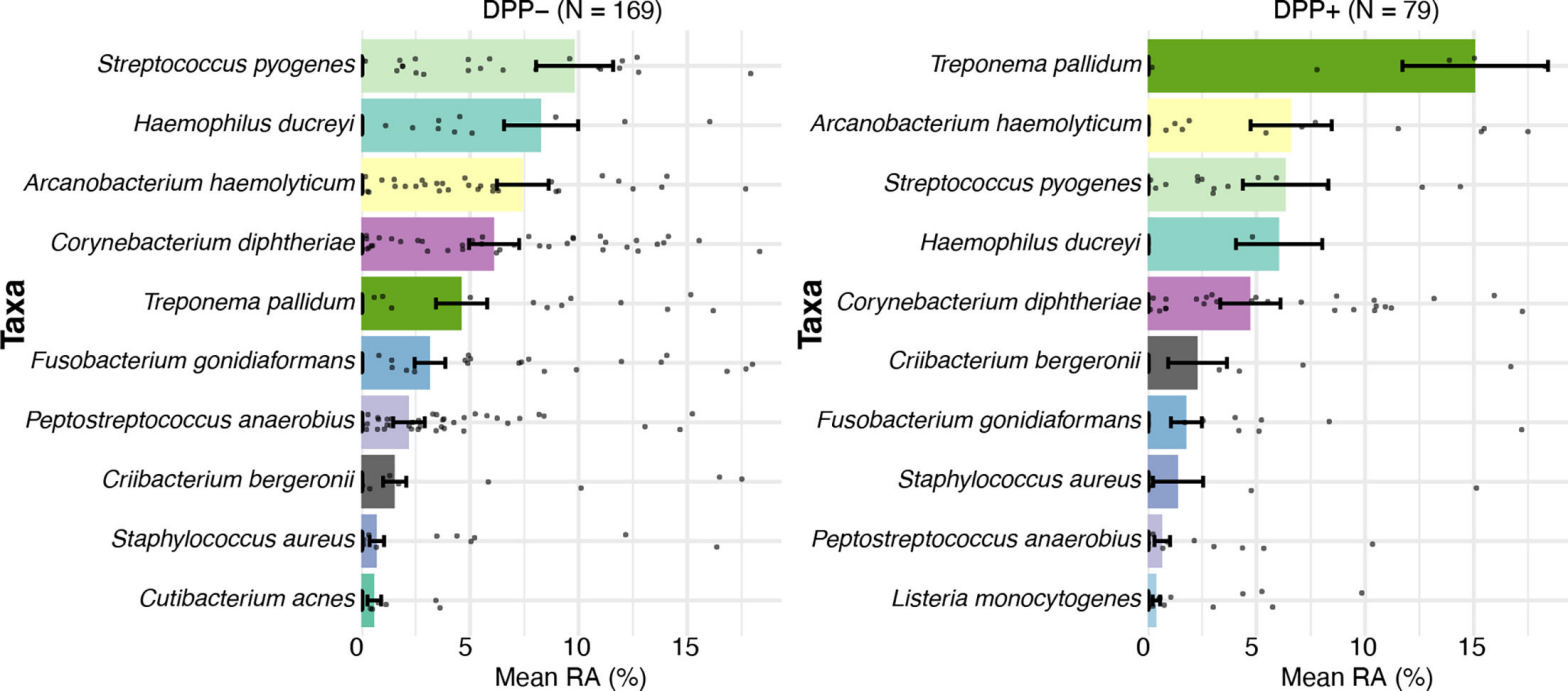
The top 10 most relatively abundant bacterial taxa in CU of DPP+ vs. DPP− participants. The relative abundances of ALR-transformed MetaPhlAn3 counts for bacteria were categorized according to the result of the DPP test. The dots represent the relative abundance of specific taxa in individual samples. CU, cutaneous ulcer; DPP, Dual Path Platform; RA, relative abundance.

We next compared the most relatively abundant bacteria in specimens from DPP+ vs. DPP– participants (Fig. 3). The top nine bacterial species were the same in both groups, but their abundances differed (Fig. 3). For example, TP had a mean relative abundance of 15.07% in DPP+ specimens vs. 4.61% in DPP– specimens, and this difference was significant by *t*-test (*P* = 0.02) but not significant by Wilcoxon test (*P* = 0.23) or after adjustment for multiple comparisons (adjusted *P* > 0.4). These data suggest that the children whose CU had lower TP relative abundance (e.g., early yaws) may not have developed antibodies to the organism at the time they were sampled. In contrast, the relative abundances of other species of interest, *A. haemolyticum* (6.6% vs. 7.4%), *C. diphtheriae* (4.7% vs. 6.1%), *C. bergeronii* (2.3% vs. 1.5%), *F. gonidiaformans* (1.8% vs. 3.2%), and *Peptostreptococcus anaerobius* (0.67% vs. 2.2%), were similar between the DPP+ and DPP– groups (all adjusted *P* > 0.4; Table S1). There were no significant differences in ⍺-diversity between DPP+ vs. DPP− specimens (data not shown). However, β-diversity differed significantly when measured using either Bray-Curtis distance (*P* = 0.035) or Euclidean distance on the ALR-transformed count data (*P* = 0.005) by PERMANOVA.

### Enriched bacterial species in IU

**Fig 4:**
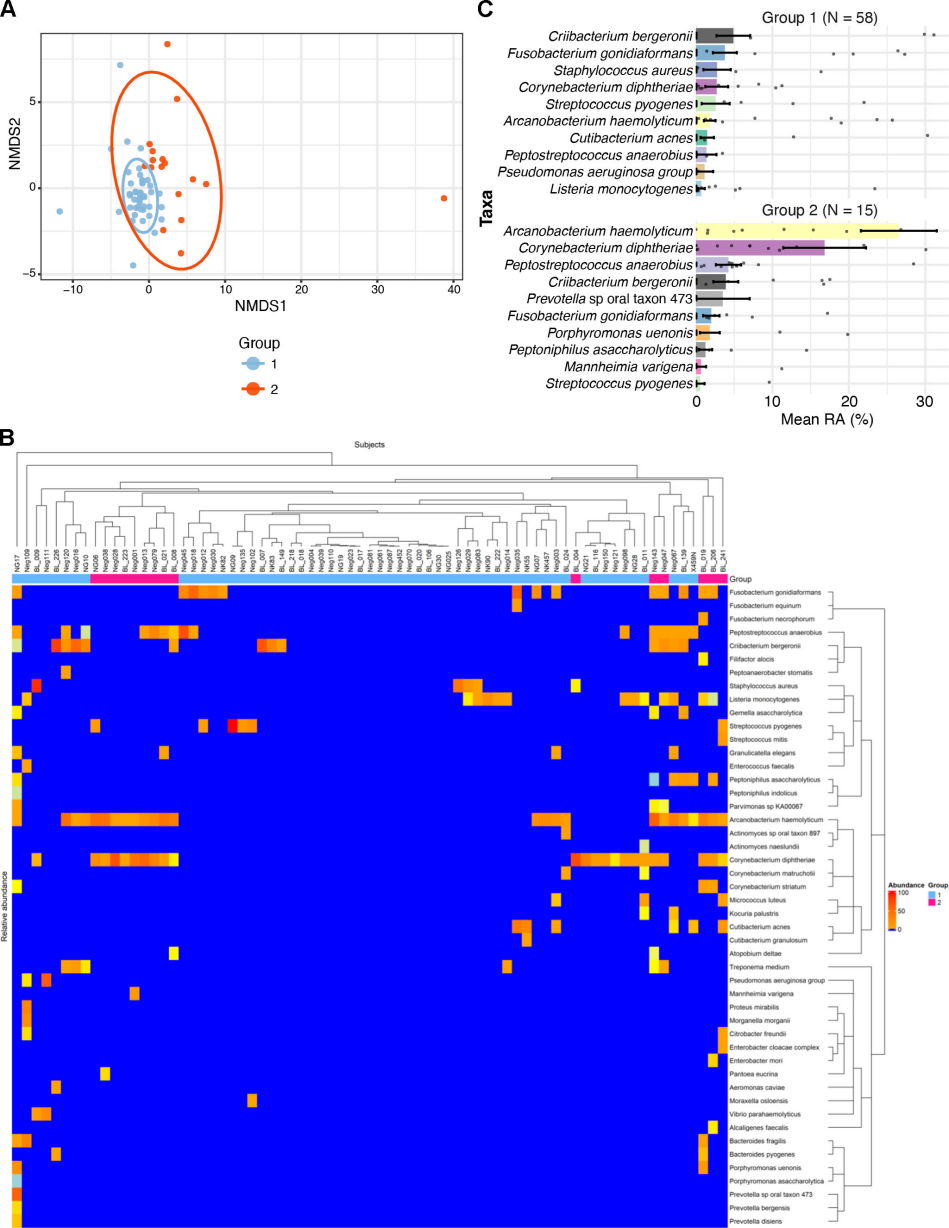
IUs form two groups. (**A**) NMDS plot of ALR-transformed bacterial communities from IU group 1 and group 2. Ordination was computed using Euclidean distances, and 95% confidence ellipses are shown for each group. Group structure differed significantly by PERMANOVA (*P* = 0.001). (**B**) Heatmap of the relative abundance of each organism across specimens. (**C**) The top 10 most relatively abundant bacteria in each IU group. RA, relative abundance.

We next asked whether there were highly abundant bacterial species that were associated with IU. Four of the 77 IU specimens contained no microbial reads and were excluded from the analysis. Using the Calinski-Harabaz algorithm and silhouette analysis, we clustered the bacterial microbiomes of the remaining 73 IU specimens into two groups that had significantly different overall bacterial community structures by PERMANOVA (*P* = 0.001; Fig. 4A). Group 1 contained 58 specimens, and group 2 contained 15 specimens (Fig. 4B). Group 2 specimens were dominated by two species, *A. haemolyticum* and *C. diphtheriae*, both of which had higher relative abundances than in group 1 (*P* < 0.001); in contrast, bacteria in group 1 were more uniformly distributed than in group 2, and several taxa had similar abundances in both groups (Fig. 4C).

## DISCUSSION

Until recently, exudative CUs in tropical regions were believed to be caused exclusively by TPE, the causative agent of yaws. In the past decade, however, CUs have been shown to contain a high relative abundance of other pathogens, including HD and SP. Because our prior metagenomic studies were performed in a PNG community after MDA of AZ (13, 14), it was unclear whether our previous findings were affected by antibiotic pressure or were generalizable to other populations. Here, we show that TPE, HD, and SP are also the major pathogens enriched in CU in a second PNG community whose inhabitants had not undergone intensive antimicrobial treatment, suggesting that our initial findings were not affected by antibiotic pressure. In addition, the fact that the initial studies included specimens collected in 2016 and 2017, while the current study included specimens collected in 2023, strongly supports the generalizability of these results to PNG.

Because TPE, HD, and SP DNAs are not detected in all CU cases in PNG, we used shotgun metagenomics to investigate additional potential causative agents of IU. We identified two IU groups: one without a dominant species, suggesting a polymicrobial origin, and one dominated by high relative abundances of *A. haemolyticum* and *C. diphtheriae*. However, the latter organisms were also common and had similarly high relative abundances in TPE+, HD+, and SP+ ulcers. The overall taxonomic composition of both IU groups was broadly similar to ulcers attributed to TPE, HD, and/or SP. These findings raise two possibilities that cannot be resolved with the present data: (i) IU may represent ulcers in which an initial causative pathogen (TPE, HD, and/or SP) has already been cleared by the host immune response, or (ii) IU-associated organisms such as *A. haemolyticum* and *C. diphtheriae* may themselves be primary causes of CU rather than colonizers of pre-existing lesions. A limitation of this study is that we did not collect clinical data on ulcer chronicity or whether the ulcer was treated prior to sample collection (e.g., saliva application or topical therapies). Future studies that include these clinical data and that measure host antibody responses to *A. haemolyticum* and/or *C. diphtheriae* will be needed to distinguish colonization from causation.

We and others previously reported that in addition to HD, SP, and TPE, several other bacterial species were among the most abundant organisms in CU from children on Lihir Island after MDA of AZ, including *A. haemolyticum*, *C. diphtheriae*, *Fusobacterium gonidiaformans*, and *C. bergeronii* (14, 28). These organisms were also present in CU from children residing on New Britain Island at similar relative abundances. The data suggest that MDA of AZ also did not select for these organisms in the previous studies.

*A. haemolyticum* is a gram-positive bacillus primarily associated with pharyngitis, skin, and soft tissue infections, but it can occasionally cause invasive infections (29–32). *C. diphtheriae*, another gram-positive bacillus, is usually associated with respiratory infections but has also been detected in painful skin ulcers, where it is thought to be an opportunistic pathogen that infects pre-existing skin lesions (33). In a study using 16S rRNA gene sequencing of residual CU specimens from children in Ghana and the Solomon Islands, *A. haemolyticum* and *C. diphtheriae* were detected in 34% and 21% of the specimens, respectively, indicating that our identification of these organisms is generalizable to other geographic regions (34).

*Listeria monocytogenes* was among the top 10 bacteria detected in SP+ and SP+TPE+ samples (Fig. 2). *L. monocytogenes* causes sepsis and meningitis in neonates, immunocompromised hosts, and the elderly. *L. monocytogenes* also commonly colonizes the GI tract of ruminants such as cows, sheep, and goats. As these children live admixed with domestic farm animals, are not of the age that is at risk for sepsis, and were otherwise healthy except for having an ulcer, *Listeria* found in these CU specimens likely represents fecal contamination.

The DPP lateral flow rapid test for treponemal antibodies was used in the LZD vs. AZ trial to identify children who were likely to have yaws. Using PCR as the standard for detecting TPE DNA, the dual-positive DPP test had a sensitivity of 38.8% and a specificity of 80% for infection with TPE. Thus, ~61% of children with a dual-positive DPP test had no evidence of active TPE infection, indicating that their positive DPP test results likely were due to past or latent infections with TPE. Conversely, ~20% of children with a negative DPP test had active infections with TPE. Although only statistically significant by an unadjusted *t*-test, specimens obtained from children who were DPP− had a lower relative abundance of TPE than specimens obtained from children who were DPP+ (Fig. 3), suggesting that the DPP test may not detect children with early yaws.

LZD has a broad spectrum of activity against gram-positive organisms, such as SP, and has activity against TPE and gram-negative anaerobes, such as *Peptostreptococci* and *Fusobacteria* (35–37). LZD has borderline activity against *Haemophilus influenzae* but no activity against most gram-negative aerobes (35–37). There are no data on the susceptibility of HD, a gram-negative facultative anaerobe, to LZD. CU specimens from 14 of the 80 children who had a dual-positive DPP test contained HD DNA; if HD is not susceptible to LZD, this raises the possibility that empiric initial treatment of DPP+ children with LZD would have a failure rate of ~17.5%. However, if LZD is used only after AZ treatment failure for suspected AZ-resistant yaws, HD susceptibility to LZD would not be a concern.

In conclusion, we found that HD, SP, and TPE were enriched in CU in a second community in PNG whose inhabitants were not heavily exposed to AZ, suggesting that AZ treatment did not lead to a shift in CU-associated bacteria and supporting the generalizability of these etiologies within PNG. Currently, the World Health Organization classifies 16 countries as yaws-endemic (https://www.who.int/data/gho/data/indicators/indicator-details/GHO/status-of-yaws-endemicity). PCR-based surveys examining whether pathogens other than TPE contribute to exudative CU have been performed in only five of these countries (38), and metagenomics studies have been performed only on specimens obtained from PNG, Ghana, and the Solomon Islands (13, 14, 28, 34). This limited data represents a major gap in our understanding of the global epidemiology of exudative CU in children living in the tropics and underscores the need for further investigation.

## Data Availability

Raw sequences, raw counts, and metadata were deposited in the Sequence Read Archive with the accession number PRJNA1369741.

## SUPPLEMENTAL MATERIAL

Data set S1 msphere.00919-25-s0001.xlsx Count data and statistics for microbial reads.

Table S1 msphere.00919-25-s0002.xlsx Relative abundances in DPP+ vs. DPP- samples.

ASM does not own the copyrights to Supplemental Material that may be linked to, or accessed through, an article. The authors have granted ASM a non-exclusive, world-wide license to publish the Supplemental Material files. Please contact the corresponding author directly for reuse.
